# Unusual migration of a tooth root into the ethmoid sinus after dental extraction: a case report and literature review

**DOI:** 10.1093/jscr/rjaf737

**Published:** 2025-09-13

**Authors:** Sharif Almatrafi

**Affiliations:** Department of Otorhinolaryngology-Head and Neck Surgery, College of Medicine, Prince Sattam Bin Abdulaziz University, Aljami'ah District, Abdullah Bin Amer Street, Alkharj 16273-7201, Saudi Arabia

**Keywords:** ethmoid sinus, foreign body, dental extraction, tooth root, chronic sinusitis

## Abstract

Iatrogenic foreign bodies of dental origin are rare and frequently involve the maxillary sinuses. We report an unusual case of tooth-root migration into the ethmoid sinus after dental extraction. A 54-year-old woman with a history of maxillary left molar extraction 6 months prior presented with symptoms of left nasal obstruction, green malodorous nasal discharge, hyposmia, and left-sided facial pressure for 3 months. She was diagnosed with unilateral sinusitis, and nasal endoscopy revealed severe left middle meatus edema with thick pus discharge. Computed tomography of the paranasal sinuses revealed a radiopaque foreign body in the left anterior ethmoid sinus with complete opacification of the left paranasal sinuses. The tooth root was extracted transnasally via functional endoscopic sinus surgery. Complete symptom resolution was achieved postoperatively. Sinonasal foreign bodies can cause sinusitis owing to mucosal irritation. Endoscopic extraction is an optimal treatment owing to its short operative time and reduced perioperative morbidity.

## Introduction

Iatrogenic foreign bodies of dental origin are rare. The teeth are connected by their roots to the maxilla; hence, the maxillary sinus is most frequently involved [1]. During dental procedures, a tooth or dental filling material may be dislodged into the maxillary sinus. In most cases, they gain entry through an oroantral communication and remain in the maxillary sinus [2]. Herein, we report an extremely rare case of tooth-root migration into the ethmoid air cells following dental extraction.

## Case report

**Figure 1:**
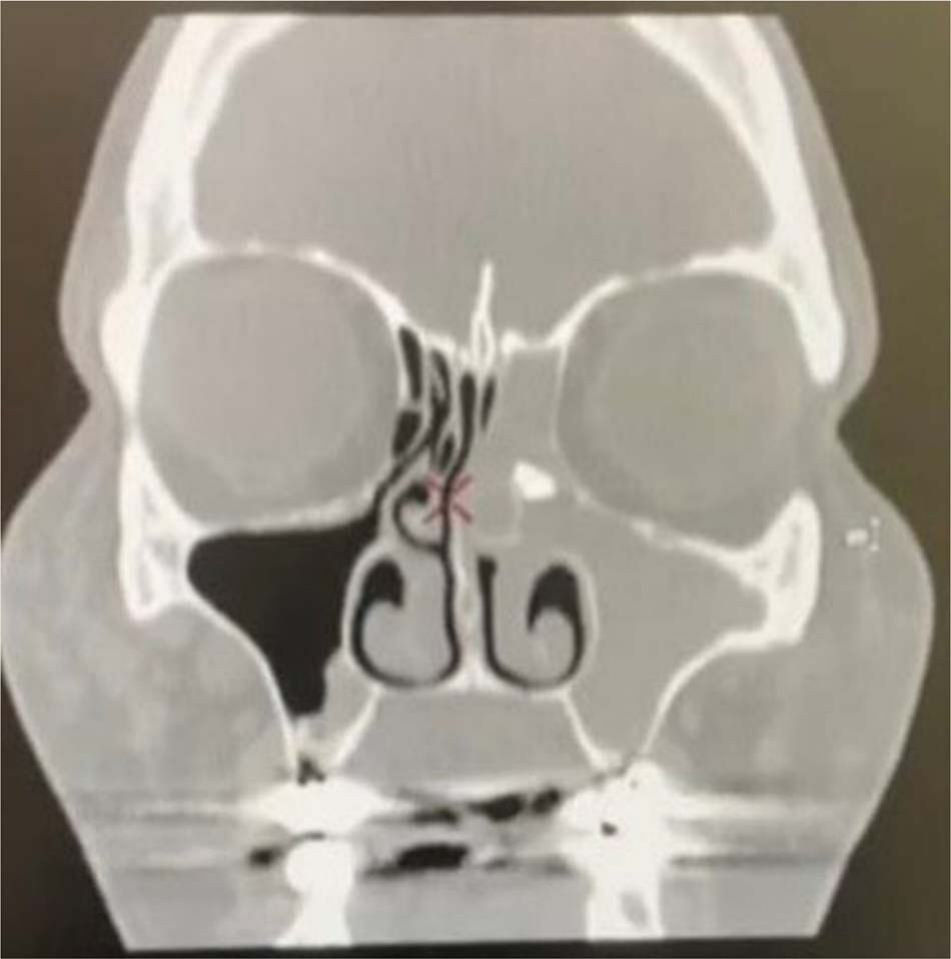
Coronal computed tomography image showing left maxillary and ethmoid sinusitis and a foreign body in the left anterior ethmoid sinus.

A 54-year-old woman with a history of extraction of the left maxillary molar tooth ⁓6 months prior visited our clinic with complaints of left nasal obstruction, green malodorous nasal discharge, hyposmia, and left-sided facial pressure for 3 months. She had been treated with multiple courses of oral antibiotics, intranasal corticosteroids, and saline nasal irrigation without improvement. The patient had a history of routine dental extraction performed by a general dentist. However, displacement of a root fragment was not documented. Nasal endoscopy on presentation revealed severe left middle meatus edema with thick pus discharge, and nasopharyngeal examination revealed post-nasal discharge. Examination of the ear, throat, neck, and cranial nerves revealed no significant findings. Computed tomography (CT) of the paranasal sinuses revealed a 10 × 6 mm radiopaque lesion in the left anterior ethmoid sinus with complete opacification of the left paranasal sinuses (Fig. 1).

**Figure 2:**
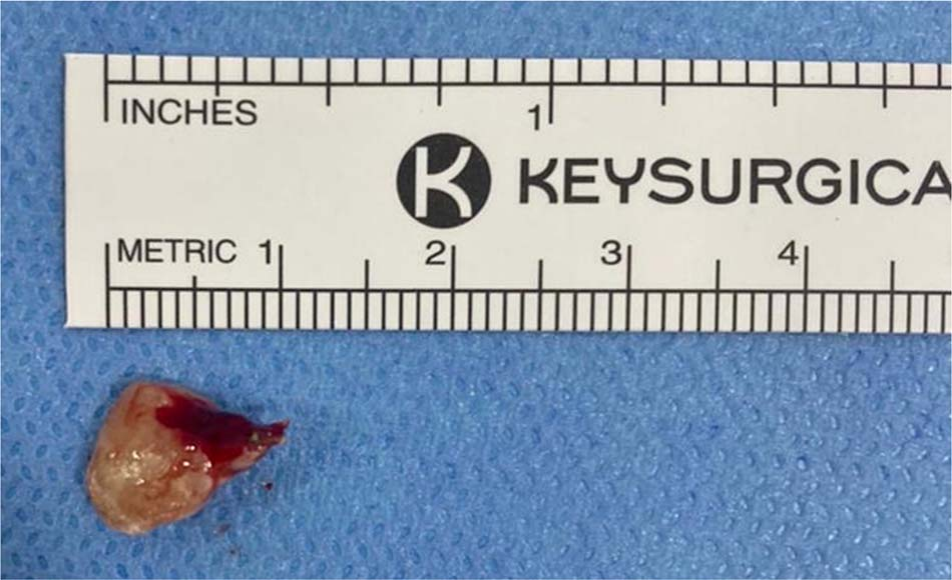
Tooth root extracted from the left anterior ethmoid sinus during endoscopic sinus surgery.

The patient underwent functional endoscopic sinus surgery with tooth-root extraction (Fig. 2). The surgery was uneventful, and the patient was discharged on the same day without any perioperative complications. Postoperatively, the patient’s symptoms resolved completely. Regular follow-up visits to the clinic with serial nasal endoscopic examinations showed clear sinuses with no evidence of disease recurrence up to 1 year after surgery.

## Discussion

Sinonasal foreign bodies are rare, are most frequently found in the nasal cavity or maxillary sinus, and are frequently associated with a history of dental procedures [1]. Therefore, asking patients about a history of dental procedures is important. Foreign bodies of dental origin typically remain in the maxillary sinuses, and several cases of tooth or implant displacement into the maxillary sinus have been reported [3–6]. This case highlights an extremely rare possibility of migration of a tooth root into the ethmoid sinus following dental extraction. Considering the integrity of the medial maxillary wall on CT, the dental root fragment may have reached the ethmoid sinus via mucociliary clearance by the columnar epithelium in an effort to drain the maxillary sinus [7].

Because foreign bodies may potentially harm the paranasal sinus mucosa and cause chronic inflammation, their detection is crucial. CT is considered the gold standard diagnostic modality for all paranasal-sinus pathologies, particularly for radiopaque lesions [8].

A thorough search of the PubMed database using the keywords ‘ethmoid sinus,’ ‘tooth,’ ‘migration,’ and ‘dental implant’ identified only six cases of dental foreign bodies in the ethmoid sinus [7–12]. Table 1 displays the primary comparisons between previously documented cases and our case. In all previously reported cases, CT was the modality of choice and surgical removal resulted in significant symptomatic improvement. Extracted foreign bodies included teeth, implants, and dental filling materials.

**Table 1:** Summary of studies reporting abnormal migration of dental foreign bodies into ethmoid sinus.

**Study (reference)**	**Case number**	**Imaging modality**	**Foreign body size**	**Surgical approach**	**Type of foreign body**	**Location**
Ishikawa, Mizuno, Yamazaki, Satoh, Notani & Fukuda 2004 [9]	1	CT	15 mm	Endoscopic transnasal	Dental filling material (gutta percha)	Left ethmoid
Shishegar, Bayat & Kazemei 2009 [10]	2	CT	Not reported	Endoscopic transnasal	Tooth	Right ethmoid
Bakhshalian, Sim, Nowzari, Cha & Ahn 2015 [11]	3	CT	Not reported	Endoscopic transnasal	Dental implant	Right ethmoid
Wong, Virk & Magarey 2021 [8]	4	CT	12 × 10 mm	Endoscopic transnasal	Tooth	Right ethmoid
Nyer 2012 [12]	5	CT	18 mm	Endoscopy-assisted lateral antrostomy	Dental filling material (gutta percha)	Right ethmoid
Lee, Yoon, Lee & Lim 2019 [7]	6	CT	Not reported	Endoscopic transnasal	Dental implant	Left ethmoid
Present case	7	CT	10 × 6 mm	Endoscopic transnasal	Tooth	Left ethmoid

Sinonasal foreign bodies can persist for a long time without causing symptoms. However, in symptomatic patients, persistent mucosal irritation and ciliary dysfunction can lead to chronic sinus inflammation. The typical presentation is unilateral sinusitis, with symptoms such as nasal obstruction, malodorous nasal discharge, and facial pain. In most symptomatic patients, surgical extraction results in complete symptom resolution. However, removal of sinonasal foreign bodies is generally advised even before symptom onset [13]. In this case, root-fragment displacement was likely not recognized at the time of extraction. This highlights a key learning point: retained root fragments can go unnoticed during dental extractions. Prompt identification and retrieval, or referral to an oral or maxillofacial surgeon, is crucial to prevent complications such as migration or chronic sinusitis [14].

## Conclusion

Sinonasal foreign bodies, particularly in the ethmoid sinuses, are rare and can cause sinusitis owing to mucosal irritation. Patients typically present with unilateral nasal obstruction, malodorous discharge, and facial pain. Careful extraction techniques and vigilance during dental procedures, combined with immediate investigation of suspected displacement, can reduce the risk of iatrogenic foreign body complications. Early referral to an oral or maxillofacial surgeon is advised when retrieval cannot be safely performed. CT is the modality of choice for evaluating sinusitis and detecting sinonasal foreign bodies of dental origin. Endoscopic extraction is the procedure of choice owing to its short operative time and reduced perioperative morbidity. However, the limitations of this approach include the need for specialized equipment and training [6]. Regular follow-up with serial endoscopic examinations is required to ensure the resolution of sinus inflammation.
